# Functional Gene Group Analysis Indicates No Role for Heterotrimeric G Proteins in Cognitive Ability

**DOI:** 10.1371/journal.pone.0091690

**Published:** 2014-03-13

**Authors:** W. David Hill, Christiaan de Leeuw, Gail Davies, David Cherry McLachlan Liewald, Anthony Payton, Leone C. A. Craig, Lawrence J. Whalley, Mike Horan, William Ollier, John M. Starr, Neil Pendleton, Danielle Posthuma, Timothy C. Bates, Ian J. Deary

**Affiliations:** 1 Centre for Cognitive Ageing and Cognitive Epidemiology, University of Edinburgh, Edinburgh, United Kingdom; 2 Department of Psychology, University of Edinburgh, Edinburgh, United Kingdom; 3 Center for Neurogenomics and Cognitive Research, Neuroscience Campus Amsterdam, Complex Trait Genetics, VU University Amsterdam, Amsterdam, The Netherlands; 4 Institute for Computing and Information Sciences, Radboud University Nijmegen, Nijmegen, The Netherlands; 5 Centre for Integrated Genomic Medical Research, University of Manchester, Manchester, United Kingdom; 6 Public Health Nutrition Group, Institute of Applied Health Sciences, University of Aberdeen, Aberdeen, United Kingdom; 7 Institute of Applied Health Sciences, University of Aberdeen, Aberdeen, United Kingdom; 8 Centre for Clinical and Cognitive Neurosciences, Institute of Brain, Behaviour and Mental Health, University of Manchester, Manchester, United Kingdom; 9 Department of Clinical Genetics, VU University Medical Center, Amsterdam, the Netherlands; 10 Department of Child and Adolescent Psychiatry, Erasmus University Rotterdam, Sophia Child Hospital, Rotterdam, The Netherlands; Institut Jacques Monod, France

## Abstract

Previous functional gene group analyses implicated common single nucleotide polymorphisms (SNPs) in heterotrimeric G protein coding genes as being associated with differences in human intelligence. Here, we sought to replicate this finding using five independent cohorts of older adults including current IQ and childhood IQ, and using both gene- and SNP-based analytic strategies. No significant associations were found between variation in heterotrimeric G protein genes and intelligence in any cohort at either of the two time points. These results indicate that, whereas G protein systems are important in cognition, common genetic variation in these genes is unlikely to be a substantial influence on human intelligence differences.

## Introduction

People who do well on one type of cognitive test tend to do well on others, giving rise to the concept of general cognitive ability (intelligence) as an important human phenotype [1]. The strong heritability [2], and significant practical impact of general intelligence on factors such as education, occupational status, and health, has motivated research seeking to discover molecular genetic factors influencing cognitive differences [3]. However, specific genetic variants remain elusive, other than a small effect of *APOE* variation on cognitive ageing [4]. The failure of candidate gene designs to identify genetic variants being involved in intelligence differences has led researchers to adopt new approaches as well as motivating the efforts dedicated to the attempted replication of reported associations [4], [5]. Methods to increase the power to detect causal variants focus on combining the effects of multiple SNPs. At the largest level, Genome-Wide Complex Trait Analysis (GCTA) can be used to combine the effect of every available SNP across the genome [6]–[8], whilst GCTA provides a heritability estimate based on all SNPs it cannot be used to detect which SNPs are associated with the trait. GCTA has been used to show that common variants jointly tag 51% of the variance in fluid cognitive ability and 40% of crystallised ability in a cohort of older adults [4], and around 40% of childhood IQ variance [9].

Pathway analyses have been introduced as a method to increase statistical power and test for the joint effect of multiple SNPs [10]. Typically, SNPs are grouped together based on their role in biological pathways or according to the cellular function of their gene product. Using this method, genetic variation within a group of genes can be examined for an association with a trait by aggregating potentially small but consistent associations with a phenotype across the gene group.

Aggregation of genes based on functional gene sets has the potential to increase power and to provide a mechanistic account of human intelligence differences [10], [11]. This is typically achieved by grouping genes according to their biological role; however, because pathways are not independent, this approach can lead to the same genes appearing in multiple pathways. Rather than grouping by “vertical” pathways, Ruano et al. [12] grouped genes according to their cellular function, an approach they termed horizontal pathway analysis. Signalling systems such as the acetylcholinergic or glutamatergic signalling pathways can be viewed as linear (vertical) pathways. However, linear pathways can share proteins, this property can be exploited for an additional increase in power by grouping genes according to their cellular function such as ligand gated ion channels, neurotransmitter metabolism, and G protein relays, as each gene set now has the potential to influence multiple linear pathways. Grouping genes across traditionally defined linear pathways, termed “horizontal grouping” by Ruano et al. [12] has the additional advantage of producing non-overlapping gene sets.

Based on a priori hypotheses, Ruano et al. [12] focussed on genes expressed in the synapse. One set was formed from synaptic-expressed genes taken both as a whole, and further divided into 17 horizontal pathways and 4 vertical synaptic signalling pathways, along with a group of genes expressed in the synapse, but whose function was unknown at the time. Among the 23 groups was a set of 27 genes coding for heterotrimeric G proteins. Heterotrimeric G proteins are activated in response to G-protein-coupled receptor binding [13]. This activation initiates an intracellular signalling cascade with effects throughout the brain. Of interest in accounting for general cognitive ability, as these same G-proteins are used in numerous synaptic signalling pathways, they potentially create a processing bottleneck which could affect a diverse range of cognition-related functions, in keeping with a role in general cognition.

Pathways were formed by Ruano et al. [12] for each available gene (Of these 27 genes (see table 2) 25 had SNP coverage in the Perlegen chip used) based on all SNPs located within the region spanning from 2 kb upstream to 500 bp downstream of the boundary of each member gene. The resulting SNP lists were tested for association using software to test for Joint Association of Genetic variants: (JAG: http://ctglab.nl/software/jag). This software uses phenotype permutation to create an empirical test for significant associations between the phenotype and the aggregated SNPs in a pathway [14]. Such tests can be either self-contained or competitive. Self-contained tests evaluate the association of SNPs in a pathway against a null hypothesis of no association. By contrast, a competitive test evaluates evidence for association in a candidate pathway against competing random selections of genes forming a baseline level of association [11]. At the time of the Ruano et al. [12] study only self-contained tests were implemented in the JAG algorithms but the software now implements competitive tests. The 23 gene sets analysed by Ruano et al. [12] were subjected to self-contained testing for association with four subtests of the Wechsler Intelligence Scale for Children in a sample of 627 children with ADHD. One gene set – the group of 25 genes (359 SNPs) coding for heterotrimeric G proteins – showed evidence for significant association: with an empirical p-value of 0.0015 against an experiment-wide α of 0.0022. This association was replicated in the UK ALSPAC cohort (n = 1,507, p = 0.047). The testing in ALSPAC differed slightly, in that two genes available in the discovery cohort – *GNB2* and *GNG11–* were omitted due to lack of coverage, with a total of 265 SNPs tested, mapped to 23 of the 25 genes used in the discovery cohort. G protein coding genes, then, may be causally associated with intelligence, accounting for around 3.3% of variance in general ability [12].

Here, we sought to replicate the association of G protein coding genes with intelligence. As in the original Ruano et al. [12] method, we used the self-contained option in JAG. In addition we used an alternative method – Gene Set Enrichment Analysis (GSEA [10], [11], [15]– as a complementary analysis strategy. GSEA works with gene-level association statistics created by programs such as Versatile Gene Analysis System (VEGAS [16]) and performs a competitive test of enrichment to determine if genes within the candidate pathway show a greater association to a phenotype than do equivalent sets of genes selected at random from outside the pathway. Use of both self-contained and competitive methods of analysis provide a robust test of the original hypothesis that variation in heterotrimeric G proteins is associated with general cognitive ability. In addition, due to the longitudinal nature of the Lothian and Aberdeen cohorts [17] we were able to test for association both with current cognitive ability in older adults in five independent samples, and also for childhood (age 11) IQ scores in two of these samples. With α set at 0.05, a simulation-study of the power of JAG to detect the original reported-effect of 3.3% of total variation in our five-cohort meta-analysis lay between 0.78 and 0.87 depending on assumptions about the distribution of effects across the total set of SNPs in the pathway (see R-scripts for JAG power analyses in scripts S1–S2 for detail on computing power, and assumptions regarding the distribution of effect across SNPs in a pathway). Of course, given the winner’s curse, the likely true effect of the gene set, if replicable, is likely less than this.

## Materials and Methods

### Participants

Data from a total of 3,511 healthy middle-aged and older individuals were used. They form the CAGES consortium (Cognitive Ageing and Genetics in England and Scotland) of five cohorts. These cohorts include the Lothian Birth Cohorts of 1921 and of 1936 (LBC1921, LBC1936) [18], the Aberdeen Birth Cohort of 1936 (ABC1936) [17], and the Manchester and Newcastle Longitudinal Studies of Cognitive Ageing Cohorts [19].

LBC1921 consists of 550 (316 female) individuals, most of whom took part in the Scottish Mental Survey 1932 (SMS1932) [20], [21], [22]. These individuals were generally healthy and living independently in Edinburgh city and the surrounding Lothian region [21], and recruited to the LBC1921 study in 1999–2001. The mean age at recruitment was 79.1 years (SD = 0.6). Identification of potential participants was carried out using the records of those registered with a general practitioner in the area and by media advertisements. DNA was extracted from venesected whole blood collected following informed consent. All subjects gave written consent and ethical approval was granted by The Lothian Research Ethics Committee.

LBC1936 consists of 1091 (543 female) individuals, most of whom took part in the Scottish Mental Survey 1947. These individuals were generally healthy and living independently in Edinburgh city and the surrounding Lothian region [23]. Between in 2004 to 2007 at about age 70 they were first recruited to the LBC1921 study. The mean age at recruitment was 69.5 years (SD = 0.8). Identification of potential participants was carried out using the records of those registered with a general practitioner in the area and by media advertisements. DNA was extracted from venesected whole blood collected following informed consent. All subjects gave written consent and ethical approval was granted by Scotland’s Multicentre Research Ethics Committee and the Lothian Research Ethics Committee.

Those in ABC1936 were drawn from the original members of Scottish Mental Survey 1947. These 498 (255 female) individuals, living in the Aberdeen area, were recruited at a mean age of 64.6 (SD = 0.9) years between 1999 and 2003. Each member of ABC1936 was relatively healthy and all lived independently in the community [17]. Following informed consent, each member had DNA samples collected from venesected whole blood. All subjects gave written consent and The Grampian Research Ethics Committee granted ethical approval.

Participants in The Manchester and Newcastle Longitudinal study of Cognitive Ageing Cohort were assembled and tested over a 20-year period beginning in 1983/1984 [19]. This resulted in a sample size of 6,063 (4,238 female, median age = 65 years, range = 44 to 93 years) healthy participants who were living independently within the community [19]. Following informed consent, DNA was extracted from venesected whole blood taken from 805 of the Manchester cohort (572 female) and 758 of the Newcastle cohort (536 female). All subjects gave written consent and ethical approval was granted from the University of Manchester.

### Cognitive Phenotypes

Three cognitive ability phenotypes were tested for association in this study. These were general fluid cognitive ability (*g_f_*) and crystallised cognitive ability at older age (all five cohorts), and IQ at age 11 (LBC1921, LBC1936). Fluid ability describes an individual’s ability to deal with novel information [24]. Tests of fluid ability often involve reasoning tasks with novel information, detecting patterns from observations, and tests with a minimal verbal component. Fluid ability is more susceptible to the effects of normal ageing and represents the current level of cognitive functioning of our sample. Crystallised ability describes the capacity to apply previously learned information to a problem; it is the level of knowledge an individual has acquired over their lifetime [24]. It is typically assessed by tests of vocabulary, reading ability or general knowledge. Crystallised ability is less susceptible to the effects of ageing [25] and is therefore used here as an indicator of the participants’ peak intellectual capacity prior to the ageing process, potentially an important factor as the participants tested here are older than those used in Ruano et al. [12]. The general fluid ability factor was derived using different test batteries in each of the cohorts. The high correlations known to exist between general factors extracted using different test batteries strongly indicate they are measuring the same underlying trait [26] and so are comparable between batteries/cohorts.

The *g_f_* factors were extracted separately for each cohort based on available tests. In LBC1921, four tests were used: the Moray House Test [21], Raven’s Standard Progressive Matrices [27], phonemic verbal fluency [28], and Wechsler Logical Memory scores [29]. Six non-verbal tests were used to derive *g_f_* for LBC1936: Digit Symbol Coding, Block Design, Matrix Reasoning, Digit Span Backwards, Symbol Search, and Letter-number Sequencing, all components of the Wechsler Adult Intelligence Scale III^UK^ (WAIS-III^UK^) [30]. Four tests were used in ABC1936: the Rey Auditory and Verbal Learning Test (R-AVLT) [28], the Uses of Common Objects [31], Raven’s Standard Progressive Matrices [27], and Digit Symbol from the Wechsler Adult Intelligence Scale Revised (WAIS-R) [32]. In LBC1921, LBC1936 and ABC1936 the raw scores from each test were factor analysed, and Bartlett regression scores on the first unrotated component derived. To control for the effects of age, sex and population stratification, a linear regression model was run using these scores as the dependent variable, with age, sex and the first four multidimensional scaling factors (MDS) from the GWAS used as predictors. Standardised residuals from this model were extracted and retained for subsequent analyses.

In both the Manchester and Newcastle ageing cohorts, general fluid ability was based on scores on the first unrotated factor (based on maximum likelihood factor analysis) of age- and sex-residualised scores on parts 1 and 2 of the Alice Heim test 4 [33] and the four subtests of the Cattell Culture Fair Test [34]. Finally, extracting the standardised residuals from a linear model where the factor score was used as the dependent variable and the four MDS components were included as predictors controlled for the effects of population stratification.

In each of the five cohorts crystallised ability was measured using a single vocabulary test; the National Adult Reading Test (NART) [35] in the LBC1921, LBC1936 and ABC1936, and sections A and B from the Mill Hill vocabulary test [36] in the Manchester and Newcastle cohorts. Again age, sex and population stratification were controlled for by extracting the standardised residuals from a linear regression model.

Age–11 IQ was assessed using the Moray House Test (MHT) for both LBC1921 and LBC1936. The score on the MHT was corrected for age at the time of testing before being converted into an IQ-type score (mean = 100, SD = 15). Following this, sex and population stratification was controlled for by extracting standardised residuals as described in the other phenotypes presented here.

### Genotyping and Quality Control

The procedures used here for both genotyping and quality control for the five cohorts have been described elsewhere [3]. A total of 3,782 participants from the CAGES consortium were genotyped for 599,011 common single nucleotide polymorphisms (SNPs) using an Illumina610–QuadV1 chip (Illumina, Inc., San Diego, CA, USA). A total of 549,692 SNPs were retained in 3,511 subjects (2,115 females) following quality control. Unresolved gender discrepancies, relatedness or call rate <0.95, as well as evidence of non-Caucasian descent resulted in the removal of individuals from the sample. All SNPs which were included in the analysis had a call rate of >0.98, minor allele frequency of >0.01 and a Hardy-Weinberg equilibrium test of P>0.001. Multidimensional scaling analysis was carried out on the remaining individuals. The effects of population stratification on the cognitive phenotypes were controlled for by using the first four multidimensional scaling components in a linear regression. For the gene-set enrichment analysis, SNP coverage was maximised using data imputed to HapMap phase II CEU (NCBI build 36 release 22) reference panel using MACH (v1.0.16) [37].

### Candidate Gene Sets

The candidate gene set for the heterotrimeric G protein pathway tested by Ruano et al. [12] consisted of 27 genes, of which 25 were tested in the original report using a total of 359 SNPs (see their table 4). Our cohorts were genotyped on a different platform that, whereas it did not include coverage of *GNB2*, nevertheless had significantly higher coverage of the remaining genes, with a total of 473 SNPs available for testing. For GSEA, which was done using imputed SNP data, coverage of all 27 genes was achieved. The analyses thus achieve better overall coverage of the theoretically relevant trait variants in G protein genes.

### Analyses

For processing by JAG, a total of 473 SNPs were assigned to 24 genes based on their position in the UCSC Genome browser hg18 assembly with a 2 kb upstream and 500 bp downstream boundary, following the procedure in Ruano et al. [12]. The analysis was conducted with 10,000 permutations of the phenotype and an empirical p-value was derived. For GSEA, construction of the phenotypes was as described above; however, stratification was controlled via inclusion of the first four multidimensional scaling factors (MDS) as covariates in the GWAS rather than through residualisation of the cognitive phenotypes. SNPs with an imputation quality score of greater than 0.3 and a minor allele frequency of >0.005 were retained for analysis. GWAS data from the five cohorts was meta-analysed using an inverse variance weighted model in METAL [38]. Gene-based association statistics were computed on the combined GWAS using VEGAS which controls for both Linkage disequilibrium (LD) and the number of SNPs within a gene through simulation [16]. SNPs were assigned to autosomal genes according to their position on hg18 Genome Browser assembly with a ±50 kb boundary around each gene used to capture regulatory elements. The full complement of 27 genes considered to form the heterotrimeric G protein horizontal pathway were available in this analysis, which is two more than were available in the original paper due to insufficient coverage on the Perlegen chip [12]. GSEA was then used to determine if the 27 heterotrimeric G protein genes were preferentially distributed in the upper portion of each genome wide ranked gene set using a running-sum Kolomogorov-Smirnov (K-S) statistic weighted by the p-values of the gene-association statistic. These genome wide ranked set was permuted 15,000 times to derive an empirical likelihood of association, describing the proportion of observed permuted K-S tests smaller than the original weighted K-S test statistic.

### Power

**Figure 1:**
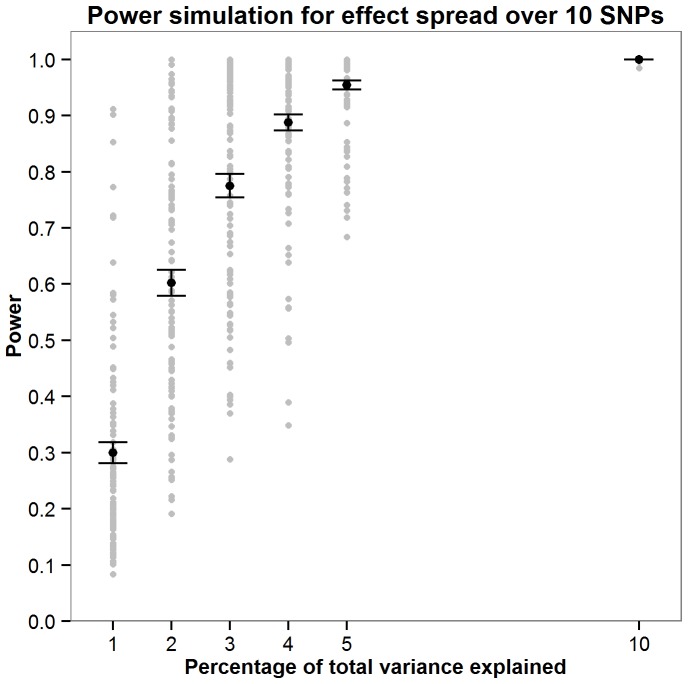
Simulations exploring how power fuctuates as a function of total variance explained when the effect is spread over 10 SNPs. For each effect size (1,2,3,4,5 or 10% of the total variance) 100 simulations were carried out. The mean power from these 100 simulations is shown in red with error bars depicting ±1 standard error.

**Figure 2:**
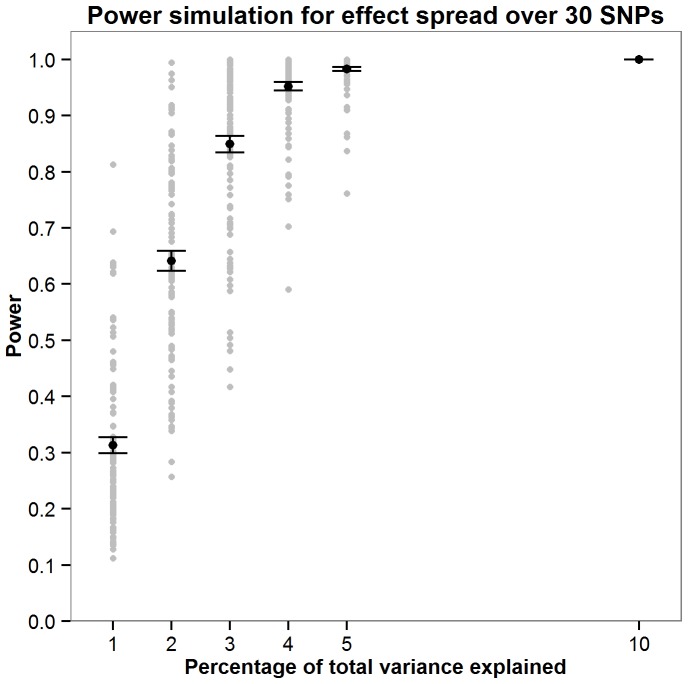
Simulations exploring how power fuctuates as a function of total variance explained when the effect is spread over 30 SNPs. For each effect size (1,2,3,4,5 or 10% of the total variance) 100 simulations were carried out. The mean power from these 100 simulations is shown in red with error bars depicting ±1 standard error.

**Figure 3:**
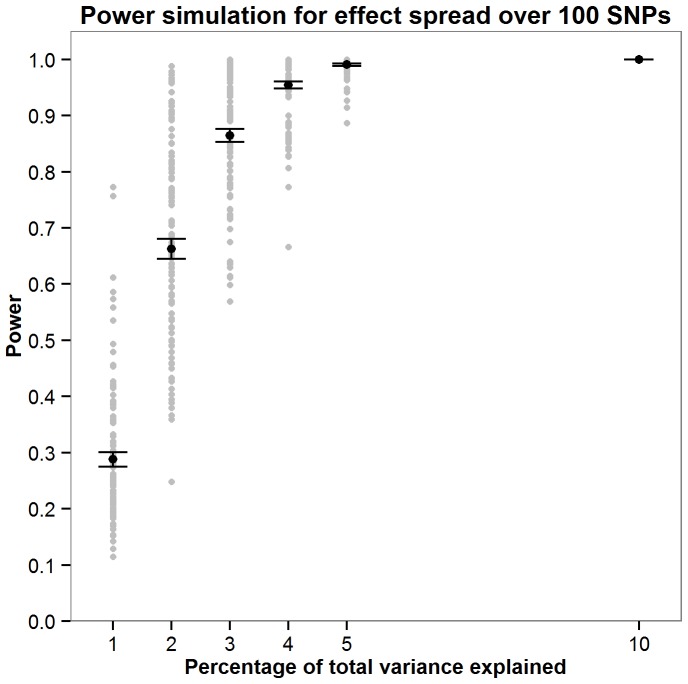
Simulations exploring how power fuctuates as a function of total variance explained when the effect is spread over 100 SNPs. For each effect size (1,2,3,4,5 or 10% of the total variance) 100 simulations were carried out. The mean power from these 100 simulations is shown in red with error bars depicting ±1 standard error.

**Figure 4:**
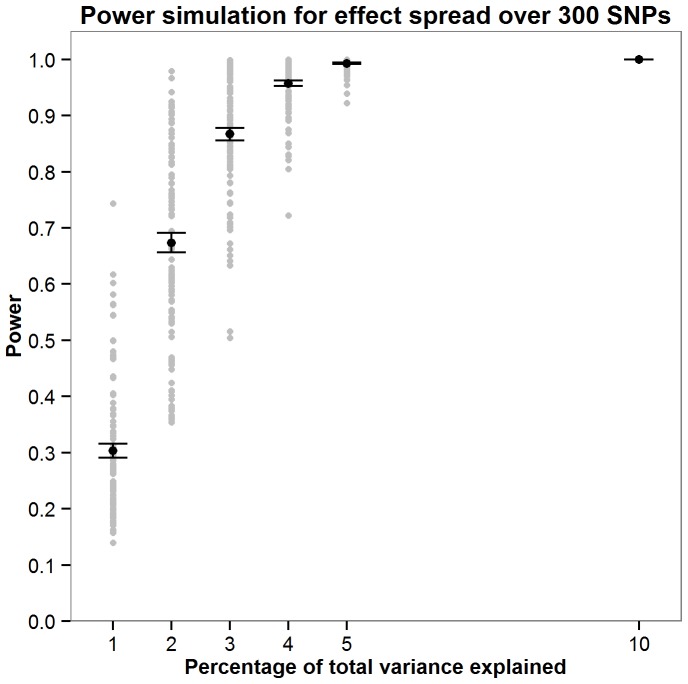
Simulations exploring how power fuctuates as a function of total variance explained when the effect is spread over 300 SNPs. For each effect size (1,2,3,4,5 or 10% of the total variance) 100 simulations were carried out. The mean power from these 100 simulations is shown in red with error bars depicting ±1 standard error.

**Figure 5:**
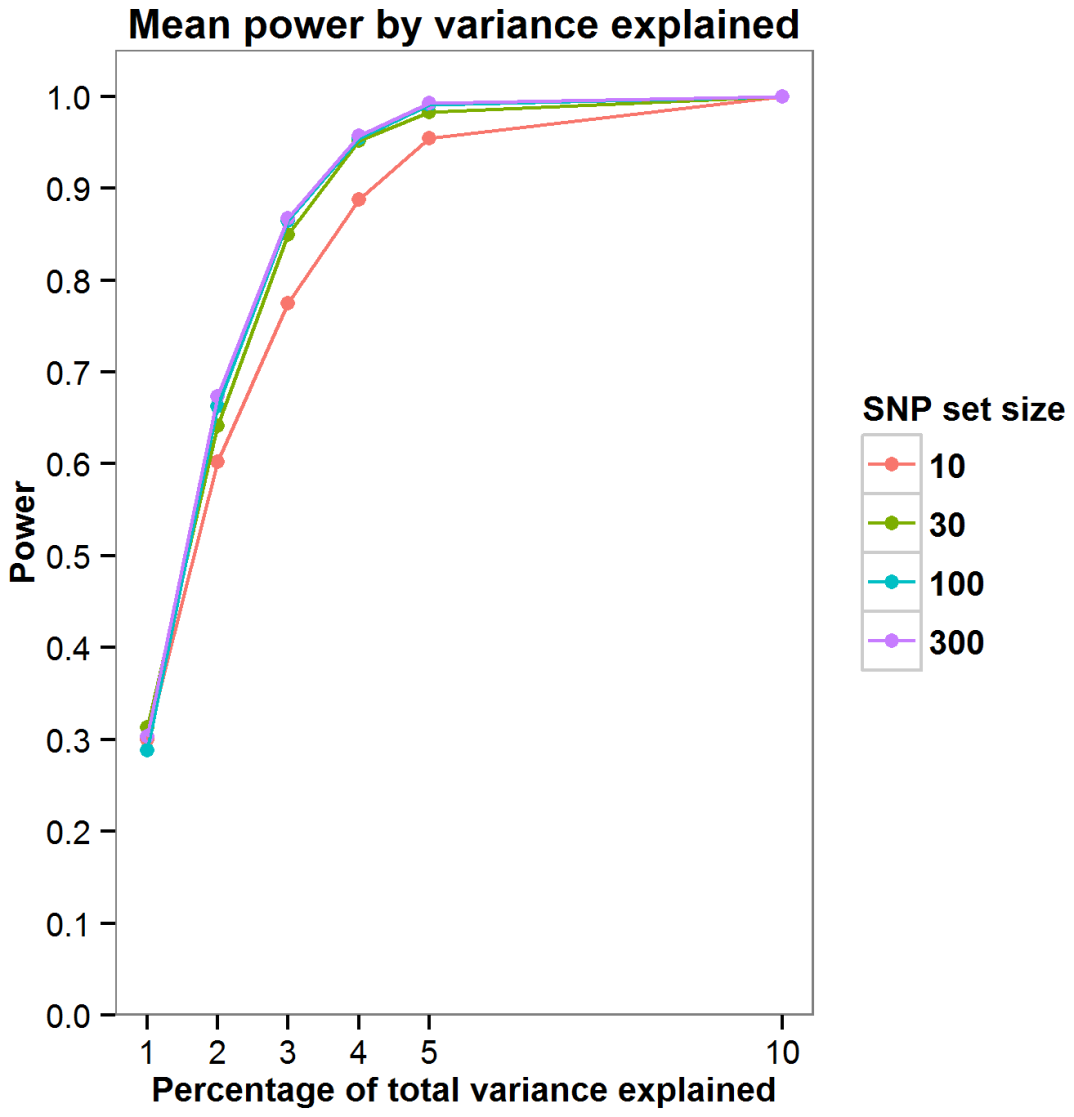
The mean power is plotted against variance explained for the number of SNPs the effect is spread over. As indicated, the total amount of variance explained, rather than the number of SNPs the effect is spread over, influences statistical power.

Power was calculated for the JAG self-contained test through simulations. As the parameters required to accurately compute power would require prior knowledge of the genetic architecture of intelligence, a series of simulations were run to explore how power fluctuates as a function of the number of SNPs the effect is distributed across and the total amount of variation this effect contributes toward. Simulations were carried out by assuming a non-zero effect for 10, 30, 100 and 300 SNPs. The percentage of variance explained by these effect SNPs was also varied where an effect size of 1, 2, 3, 4, 5 or 10% of the total variance was simulated. For the each SNPs with a non-zero effect an effect size was allocated by randomly sampling a normal distribution of effect sizes (mean = 0, SD = 1). A predicted phenotype was then calculated for each individual in each cohort using the effect sizes allocated to the effect SNPs. Next, 1000 phenotype vectors were generated within each sample by adding normally distributed noise with variance such that the predicted phenotype accounted for required percentage of total phenotypic variance (1,2,3,4,5 or 10%). For each of the 1000 phenotypes JAG was then used to calculate the gene set based p-value in each cohort. These were then meta-analysed using Stouffer’s weighted Z score to derive one p value describing the strength of the association across the five cohorts. Power was calculated as the number of the 1000 phenotypes in which the meta-analytic p-value was less than 0.05. New effect sizes were allocated to the effect SNPs 100 times as illustrated in figures 1–4 with figure 5 showing the mean power for each condition simulated. These simulations indicate that there are only negligible fluctuations in power attributable to the number of SNPs the effect is spread across. Rather, power is largely a function of the total amount of variance explained by the SNP set. The article by Ruano et al. [12] indicated that 3.3% of the variance explained could be attributed to the g protein SNP set meaning that the present study would have between 0.775 and 0.867 power to detect the effect if it was present.

## Results

None of the SNPs from the G protein groups reached genome wide significance in any of the cohorts examined (See tables S1–S12). Using JAG, tests for association between SNPs in G protein genes and either fluid or crystallised cognitive ability were non-significant in all five cohorts considered (See Table 1). Using Stouffer’s Z, weighted by the square root of the sample size [39] [40] a single meta-analytic p-value was derived for evidence of association between the gene set and the phenotype across the five cohorts. This revealed no significant evidence for association for either fluid ability (p = 0.43) or crystallised ability (p = 0.98).

**Table 1:** Association between fluid and crystallised ability and the G protein gene set for each of the five CAGES cohorts.

	Fluid ability			Crystallised ability		
Cohort	N	N SNPs	Empirical p-Value	N	N SNPs	Empirical p-Value
Lothian Birth Cohort 1921	505	468	0.68	515	468	0.66
Lothian Birth Cohort 1936	989	468	0.76	1003	468	0.97
Aberdeen Birth Cohort 1936	350	470	0.62	420	470	0.63
Newcastle	754	469	0.18	750	469	0.90
Manchester	805	469	0.20	770	469	0.57

We next conducted gene set enrichment analysis of the 5-cohorts using GSEA. These analyses also showed no significant enrichment for either fluid ability (p = 0.30) or for crystallised ability (p = 0.42). The gene based statistics conducted using VEGAS indicate that one gene was nominally significant for crystallised ability and three for fluid ability. However, these did not survive correction for the 27 tests performed (see Tables 2). These results indicate, then, that variation in the genes which code for heterotrimeric G proteins are no more associated with variation in cognitive abilities than expected by chance.

**Table 2:** Gene based analysis results for Crystallised and fluid cognitive ability.

				Gene based P-values	
Gene name	N SNPs	Start position (bp)	Stop position (bp)	Crystallised Ability	Fluid Ability
*GNA11*	69	3045407	3072454	0.280	0.340
*GNA12*	228	2734266	2850485	0.214	0.521
*GNA13*	54	60437294	60483216	0.844	0.705
*GNA14*	311	79228367	79453043	0.950	0.568
*GNA15*	88	3087190	3114766	0.640	0.561
*GNAI1*	186	79602075	79686661	0.251	0.049
*GNAI2*	32	50248650	50271790	0.541	0.684
*GNAI3*	102	109892708	109939975	0.804	0.937
*GNAL*	258	11679264	11871919	0.178	0.002
*GNAO1*	241	54782751	54948857	0.151	0.070
*GNAQ*	271	79525010	79836012	0.638	0.412
*GNAS*	127	56848189	56919645	0.717	0.752
GNAT1*	34	50204046	50208953	0.235	0.588
*GNAZ*	138	21742668	21797221	0.038	0.807
*GNB1*	52	1706588	1812355	0.815	0.795
GNB2**	35	100109310	100114728	0.677	0.956
*GNB3*	72	6819635	6826818	0.286	0.768
*GNB4*	94	180596569	180652065	0.946	0.027
*GNB5*	137	50200414	50270857	0.973	0.794
*GNG10*	130	113463681	113472347	0.236	0.907
*GNG11*	121	93388951	93393762	0.505	0.533
*GNG12*	239	67939736	68071730	0.611	0.725
*GNG2*	392	51396799	51506268	0.884	0.308
GNG3*	38	62231708	62233246	0.922	0.186
*GNG4*	148	233777607	233880677	0.751	0.755
*GNG5*	136	84736593	84744850	0.836	0.953
*GNG7*	143	2462217	2653746	0.440	0.210

Note: * indicates genes without coverage in Ruano et al. [12] ** Gene *GNB2* was incorporated into the GSEA analysis, but was not tested the JAG replication (Table 1).

We next tested for association with age-11 IQ in the two Lothian cohorts where this phenotype was available. These tests were conducted using the self-contained test in JAG. No significant evidence for association was present for either LBC1921 (n = 464, n SNP = 468, p = 0.90) or for LBC 1936 (n = 947, n SNP = 468, p = 0.70). Combined using Stouffer’s method, these p values for age 11 IQ yield a meta-analytic p-value of p = 0.88.

## Discussion

We attempted to replicate an association between variation in genes coding for G proteins and human intelligence differences [12]. Strengths of the present report include increased coverage of the G protein genes than was available for the original report, and wide range of samples, two assessed both in youth and in old age, and also use of a competitive test of association using a distinct methodology, that of gene-based gene-set enrichment analysis (GSEA). The phenotype is highly similar to that used in the original report, with an identical analysis strategy and the same software. In no cohort was any significant association found, and this remained the case under meta-analysis.

Age effects are unlikely to have led to a discrepancy between the original report and those of the current study, for three reasons: first, there is a high genetic correlation, between childhood and old age measures of *g* (0.62) [41]; second, we had available crystallised ability measures which are robust to ageing effects; and, third, we were able to directly test for association in participants using their IQ at age 11 in two samples. The null finding at both ages in the current study would indicate, then, that, across the life course, variation in heterotrimeric G proteins does not contribute more than a slight degree to individual differences in intelligence.

The original replication sample had a genetic background similar to that reported here (UK Caucasian [12]). Differences in genetic background between the present samples and those in the original report could alter the direction of association of individual SNPs. This genetic heterogeneity, however, would not affect our power to detect a significant pathway, as pathway analysis derives the sign of association for each SNP freshly in the new samples.

One significant difference between the discovery cohort and the present samples is that the discovery sample consisted of children and adolescents diagnosed with ADHD. However the ALSPAC validation sample was not drawn from a clinical population [12]. Sample differences, then, appear not to be able to account for the null finding in the present report. The initial discovery sample was very small (N = 627) and although there was replication in the much larger ALSPAC sample, this replication was only just significant. Given the null result in the current study, the original finding was likely due to sample fluctuation. In addition, only self-contained tests with correction for population stratification were applied, rather than competitive tests. Competitive tests are to be preferred over self-contained tests due to them being less susceptible to the effects of genomic inflation and due to the conservative nature of testing against genes drawn from outside the pathway.

Whereas SNP variation in heterotrimeric G proteins appears unrelated to cognitive abilities, the grouping of genes according to their cellular function, rather than in vertical pathways nevertheless has the potential to elucidate genetic mechanisms which act in, and potentially disrupt, multiple systems [12]. In addition, while G protein variation appears unrelated to normal variation in cognitive ability, the postsynaptic density per-se is rich in proteins – excitatory synapses of the human brain express over 1,500 genes and over 130 neurological and psychiatric disorders have been shown to arise from mutations in post-synaptic density genes [42], [43]. Indeed, subsets within this large number of genes, form supramolecular complexes such as the *N*-methyl-D-aspartate receptor complexes (NMDA-RC) [43], which is preferentially involved in rapid processing of information and updating of AMPA responsiveness [44] and which has been associated with normal variation in human intelligence [45]. As knowledge of biological pathways increases, so too the ability to utilise this information to aggregate the many thousands of small SNP based effects underlying intelligence [3], [9], [46] will increase, allowing testing for associations between psychological traits and candidate mechanisms.

## Supporting Information

Table S1 (CSV)

Table S2 (CSV)

Table S3 (CSV)

Table S4 (CSV)

Table S5 (CSV)

Table S6 (CSV)

Table S7 (CSV)

Table S8 (CSV)

Table S9 (CSV)

Table S10 (CSV)

Table S11 (CSV)

Table S12 (CSV)

Script S1 (R)

Script S2 (R)
